# Pre-Calving and Calving Management Practices in Dairy Herds with a History of High or Low Bovine Perinatal Mortality

**DOI:** 10.3390/ani3030866

**Published:** 2013-08-27

**Authors:** John F. Mee, Jim Grant, Cosme Sánchez-Miguel, Michael Doherty

**Affiliations:** 1 Animal and Bioscience Research Department, Teagasc, Moorepark Research Centre, Fermoy, Co Cork, Ireland; 2 Statistics and Applied Physics Department, Teagasc, Ashtown, Dublin 15, Ireland; E-Mail: jim.grant@teagasc.ie; 3 Regional Veterinary Laboratory, Department of Agriculture, Food and the Marine, Model Farm Road, Cork, Ireland; E-Mail: cosme.sanchez@agriculture.gov.ie; 4 Section of Herd Health & Animal Husbandry, School of Veterinary Medicine, University College Dublin, Dublin, Ireland; E-Mail: michael.doherty@ucd.ie

**Keywords:** bovine perinatal mortality, stillbirth, calving management, questionnaire, dairy

## Abstract

**Simple Summary:**

Mortality of full-term calves at calving is an increasing problem in dairy industries internationally. Multiple herd management factors contribute to such losses. This case-control study identified factors which differed between herds with high and low calf mortality. These included breeding, dietary, health and calving factors. It was concluded that calving, not pre-calving, management appears to be the most important area of concern in herds with high perinatal mortality. This indicates that farmers and their veterinarians need to focus on calving management when investigating such problems and when attempting to reduce losses in herds with high rates of bovine perinatal mortality.

**Abstract:**

Bovine perinatal mortality is an increasing problem in dairy industries internationally. The objective of this study was to determine the risk factors associated with high and low herd-level calf mortality. Thirty herds with a history of either high (case) or low (control) calf mortality were recruited. A herd-level questionnaire was used to gather information on management practices likely to impact bovine perinatal mortality. The questionnaire was divided into four subsections dealing with pre-calving (breeding, diet and body condition score, endemic infectious diseases) and calving factors. Most of the significant differences between case and control herds were found in calving management. For example, in case herds, pregnant cattle were less likely to be moved to the calving unit two or more days and more likely to be moved less than 12 hours pre-calving, they were also less likely to calve in group-calving facilities and their calves were more likely to receive intranasal or hypothermal resuscitation. These management procedures may cause social isolation and periparturient psychogenic uterine atony leading to dystocia, more weak calves requiring resuscitation and high perinatal calf mortality. The key finding is that calving, not pre-calving, management appears to be the most important area of concern in herds with high perinatal mortality.

## 1. Introduction

Perinatal mortality may be defined as death of the fetus or calf before, during or within 48 h after calving at full-term (>260 days) [1]. Perinatal mortality is a major problem in successful management of dairy young stock [2] and adversely affects milk production [3], reproduction and maternal survival [4]. 

Concerns have been raised both about high [5] and increasing rates of perinatal mortality particularly in Holstein heifers [6] and about ‘normalisation’ of these rates in modern dairy herds [7]. Maternal, fetal, environmental and management factors influence perinatal mortality. Some of these risk factors are well documented; dystocia [8,9], primiparity [10], age at first calving [11], twinning [12], foetal gender [13] and gestation length [14]. 

However, the reasons why some herds have more calf losses than others has received little attention in the literature; few studies have been conducted comparing herds with high and low loss rates, specifically in perinates. An investigation of risk factors for young calf mortality (1–90 days) between 60 Swedish herds with high and low loss rates found that inadequate calf serum alpha-tocopherol and beta-carotene concentrations, number of faecal pathogens and cases of diarrhoea were significantly more likely in high loss herds [15]. A Danish study comparing 28 herds with high and low young calf mortality (2–55 days) found that sociological factors such as the farm manager’s belief in whether they could influence loss rates was critical to calf health outcomes [16]. An Icelandic study of 70 farms found few differences in management practices (use of AI in heifers, better housing, concentrate feeding) between herds with high and low stillbirth rates [17]. 

In American beef herds it has been reported that calving in confinement and high dystocia rates were significantly associated with high, compared to, low calf morbidity (birth to weaning) herds [18]. In an earlier study on dairy farms in the UK comparing high and low calf mortality (0–24 h) in heifers’ calves, no differences were found in heifer measurements, body condition score, age at calving or calf size [19]. However, on high loss farms calving management differed and more heifers were assisted at calving and had dystocia indicating a significant farmer effect. 

These limited observations indicate a paucity of information on why perinatal bovine mortality differs between high and low loss herds. Hence, the objective of this study was to investigate the management factors associated with a low risk (LR) and a high risk (HR) of perinatal bovine mortality on commercial dairy farms using an observational study design. 

## 2. Materials and Methods

### 2.1. Study Design

Perinatal bovine mortality was defined as death of a fullterm (≥260 day gestation) calf before, during or within 48h after calving [1]. An unmatched case-control study design was chosen to establish the risk factors for perinatal bovine mortality in two risk groups of herds (low risk; LR and high risk; HR) recruited based on recent history of perinatal bovine mortality. The herd was the experimental unit. The study herds were selected from commercial dairy farmers in three farmer discussion groups [20] based in Munster, the main dairying region in Ireland. This sampling frame (n = 111 herds) was screened for spring-calving (January–June) herds, with more than 50 calvings/year and farmers with a history of good record keeping. Within this selection, herds were ranked on the average perinatal bovine mortality over the previous 3 years (2007–2009). Farmers with the highest and the lowest perinatal bovine mortality rankings who fulfilled these eligibility criteria were contacted by email and followed up by telephone contact to participate in the study. In total 30 farmers (15 case and 15 control) enrolled. Though some herds changed risk category after recruitment (eight LR and ten HR), their original risk category was used to classify the herds over the three years. The LR group had an average perinatal bovine mortality rate of 3.2% (0.6–4.6%) and the HR group had an average perinatal bovine mortality rate of 7.8% (6.7–12.4%). The national average perinatal mortality rate is 4.3% [9]. Herd size ranged from 61 to 520 cows, with an average of 164 cows/herd.

### 2.2. Data Collection and Analysis

Data on risk factors likely to impact perinatal mortality were collected from a pre-designed farmer questionnaire (Table 1). A written herd-level questionnaire to determine farm management practices plausibly linked to perinatal bovine mortality was drafted and piloted with two farm managers at Moorepark Research Centre to assess understanding of the questions. The redesigned questionnaire was emailed to each farmer in January 2010 and in January 2012. The questionnaire contained 17 questions grouped into four sections. Three sections dealt with pre-calving factors; breeding (heifer and cow breeds in the herd, breeds of service sires used on heifers and on cows), pre-calving diet and body condition score (forage, concentrate ration and macro and micronutrient supplementation of heifers and of cows pre-calving and body condition score of heifers and of cows pre-calving), endemic infectious diseases (recent clinical history of endemic infectious diseases, number of dogs and presence of foxes on the farm and vaccines used in heifers and in cows) and one dealt with calving management (timing of transfer of pregnant heifers and of cows to the calving facility, type of calving facility, frequency of observations for imminent calving, duration of natural calving allowed before intervention, type of calving aid used, number of personnel managing calvings, methods used to prevent milk fever and techniques used to resuscitate weak newborn calves). The topics were chosen based on management factors known to impact bovine perinatal mortality [1]. The questionnaire returns were examined prior to data entry and obvious errors indicating misunderstanding of a question were clarified. Data analysis consisted of tests of association between risk group (High and Low) and the per question responses. To facilitate comparison of preferable and non-preferable variable levels, some outcome variable levels were collapsed. Contingency tables were constructed for each year of observation and for the combined results and tested using Chi Square tests or Fisher’s Exact Test. The latter was included to cover those tables where one or more cell frequencies were ≤5. The Logistic procedure was used to generate odds ratios (OR) and their 95% confidence intervals for these tests. The final model fit was evaluated using the Hosmer and Lemeshow goodness-of-fit test. Differences were considered significant if *P* ≤ 0.05. All data editing and statistical analyses were carried out using appropriate procedures (Proc FREQ and LOGISTIC in SAS (SAS Institute Inc., Cary, NC, USA)).

**Table 1:** Risk factor questionnaire used to collect information on farm management practices relevant to bovine perinatal mortality.

		Bovine perinatal mortality (0-2 day calf death)						
Farmer name			Address.					
								
Mobile No.			Herd No.			ICBF client: Yes-No		
**Calving pattern:** Spring, Spring and Autumn, Autumn, Other (Circle one)								
			**Heifers**			**Cows**		
**Breeds: **								
								
**Sire breeds used on: **								
								
**Heifer feeding and minerals pre calving:**								
								
								
**Body condition score heifers precalving:** thin (<3), good (3-3.5), fat (>3.5)								
								
**Dry Cows feeding and minerals pre calving:**								
								
								
**Body condition score cows precalving:** thin (<3), good (3-3.5), fat (>3.5)								
								
**Month of Vaccinations: **			**Heifers**			**Cows**		
		BVD						
		Lepto						
		Salmonella						
		IBR						
**When animals move to calving unit precalving:** ≥2 days, 1 day, 12-24h, 6-12h, point of calving								
								
**Where are animals calved:** individual pen, group pen, tie- ups, pad								
								
**How often are animals checked for signs of calving:** every >12, 6-12, 4-6, 2-4, 1-2 hours								
								
**How long do leave heifers and cows with the calfs' legs out before you assist?**								
					**Heifers:**		**Cows:**	
								
**Do you use a calving aid:** calving jack, pulley, other-specify								
								
**How many farm personnel manage calvings?**								
								
**How do you prevent milk fever:**								
								
**How do you resuscitate weak calves at birth?**								
								
**How many dogs ___________ and are there foxes________on the farm?**								
								
**Have you a recent history of BVD, Lepto, Neospora, Salmonella, IBR clinical cases: **								

## 3. Results

The significant odds ratios and 95% confidence limits for the associations between the management factor variables and the response variables (High or Low herd BPM status) in the logistic regression analysis are shown in Table 2. All of the responses for each of the four subsections of the risk factor questionnaire are shown in Table 3, Table 4, Table 5, Table 6. The response rate to the enrolment was 27% (30/111 herds) and to the questionnaire was 100%. Where differences were found between case and control herds these are highlighted in the following text under four subheadings.

**Table 2:** Associations between management factors and herd perinatal mortality status (Odds ratios, OR and 95% confidence limits, 95%CL; referent category listed first and *P*-values) in questionnaire responses in 2010 and in 2012 and in both years.

Management factor	Year	Factor level	OR	95%CL	*P*-value
Cow service sire breed	2010	AA *vs*. Je or JexHF	0.136	0.024–0.786	0.207
	Both	AA *vs.* Je or JexHF	0.162	0.049–0.542	0.038
		HF *vs.* Je or JexHF	0.375	0.143–0.984	
Heifer BCS	2010	3.25–3.5 *vs.*≥3.75	71.993	5.734–903	0.004
		≥3.75 *vs.*≤3	0.042	0.002–0.973	
	2012	3.25–3.5 *vs.*≥3.75	168.945	9.519–>999	0.002
	Both	3.25–3.5 *vs.*≥3.75	104.153	16.004–677	0.0001
		≥3.75 *vs.*≤3	0.053	0.005–0.530	
Calving unit	Both	Combination *vs.* group	10.5	1.496–73.673	0.004
Timing of transfer to calving unit	2010	0–11.9 h *vs.* >2 d	8.75	1.241–61.681	0.175
	2012	0–11.9 h *vs.* 12–23.9 h	16.667	1.361–204	0.096
		0–11.9 h *vs.* >2 d	11.667	1.527–89.121	
	Both	0–11.9 h *vs.* 12–23.9 h	12.857	2.218–74.536	0.007
		0–11.9 h *vs.* >2 d	10	2.452–40.778	
Calving personnel	Both	1 *vs.* 2	0.267	0.072–0.987	0.045
		2 *vs.*≥3	9	1.268–63.891	
Calf revival	Both	None *vs.* straw	0.075	0.007–0.757	0.004
		None *vs.* water	0.107	0.012–0.984	

### 3.1. Breeding

In HR herds (compared to LR herds) there was more likely to be use of Je or JexHF sires on cows than HF or AA sires (Table 2 and Table 3). 

**Table 3:** Responses (No.) on breeding from farmers with a high rate (HR, n = 15) and low rate (LR, n = 15) of perinatal calf mortality.

Factor	Levels ^1^	HR 2010	LR 2010	HR 2012	LR 2012	HR & LR 2010	HR & LR 2012
Heifer breed^2^	HF	15	14	15	14	29	29
	Je or JexHF	6	5	7	6	11	13
	NR, NRx or SRxHF	2	6	4	6	8	10
Heifer service sire breed	HF	12	10	12	11	22	23
	Je or JexHF	11	6	10	6	17	16
	AA	5	8	6	9	13	15
	NR	4	6	4	6	10	10
	GS	1	0	1	0	1	1
Cow breed	HF	15	15	15	15	30	30
	Je or JexHF	4	8	6	8	12	14
	NR, SR or SRx or NRxHF	3	6	3	6	9	9
	Mo or MoxHF	0	2	0	2	2	2
Cow service sire breed	HF	14	13	14	13	27	27
	Je or JexHF	11	4	12	4	15	16
	NR	5	4	5	6	9	11
	AA	3	8	4	7	11	11
	Other beef	1	1	2	1	2	3

^1^ The values in each cell may vary between 0 and 15 for individual years and between 0 and 30 for combined years; ^2^ Breeds: AA = Aberdeen Angus, BB = Belgian Blue, GS = Murray Grey, He = Hereford, HF = Holstein-Friesian, Je = Jersey, Mo = Monbeliarde, NR = Norwegian Red, SR = Swedish Red.

### 3.2. Diet and Body Condition Score

In HR herds (compared to LR herds) there was more likely to be heifers in the target BCS range than obese heifers and more likely to be thin than obese heifers (Table 2 and Table 4). 

**Table 4:** Responses (No.) on diet and body condition score (BCS) from farmers with a high rate (HR, n = 15) and low rate (LR, n = 15) of perinatal calf mortality.

Factor	Levels ^1^	HR 2010	LR 2010	HR 2012	LR 2012	HR & LR 2010	HR & LR 2012
Heifer pre-calving diet	Grass silage	15	15	15	15	30	30
	Concentrate ration, TMR, Maize or kale	5	4	3	4	9	8
	Straw	0	4	0	3	4	3
Heifer trace element (TE) supplementation	Mineral powder	13	13	14	12	26	26
	Bolus, drench, injection or block	12	0	7	0	9	7
	No TE supplement	0	2	0	3	2	3
Heifer pre-calving BCS ^2^	3.75–4	1	12	1	13	13	14
	3.25–3.5	12	2	13	1	14	14
	≤3	2	1	1	1	3	2
Cow pre-calving diet	Grass silage	15	15	15	15	30	30
	Straw	0	5	0	3	5	3
	Concentrate ration or TMR or fodder beet	2	5	3	4	7	7
Cow trace element (TE) supplementation	Mineral powder	13	14	15	13	27	28
	Bolus, drench, injection or block	4	0	4	0	4	4
	No TE supplement	0	1	0	2	1	2
Cow pre-calving BCS	3.75–4	0	1	0	1	1	1
	3.25–3.5	14	12	14	12	26	26
	≤3	1	2	1	2	3	3

^1^ The values in each cell may vary between 0 and 15 for individual years and between 0 and 30 for combined years; ^2^ BCS = 1–5 scale.

### 3.3. Endemic Infectious Diseases

More farmers with HR herds tended to vaccinate heifers and cows against leptospirosis (*P* < 0.10), (Table 5).

**Table 5:** Responses (No.) on disease prevention, risk and occurrence from farmers with a high rate (HR, n = 15) and low rate (LR, n = 15) of perinatal calf mortality.

Factor	Levels ^1^	HR 2010	LR 2010	HR 2012	LR 2012	HR & LR 2010	HR & LR 2012
Heifer vaccination	BVD ^2^	10	10	12	9	20	21
	Leptospira	14	12	15	11	26	26
	Salmonella	12	8	14	12	20	26
	IBR ^3^	2	0	5	4	2	9
Cow vaccination	BVD	10	10	12	9	20	21
	Leptospira	14	12	15	12	26	27
	Salmonella	11	7	14	12	18	26
	IBR	2	0	5	4	2	9
Dogs on farm	Yes	12	9	12	9	21	21
	No	3	6	3	6	9	9
Foxes on farm	Yes	13	10	14	11	23	25
	Unknown	2	2	1	2	4	3
	No	0	3	0	2	3	2
Recent clinical problems	BVD	3	2	3	4	5	7
	Salmonella	5	2	5	5	7	10
	Neospora	3	0	3	1	3	4
	Leptospira	0	1	0	0	1	0
	IBR	0	0	0	1	0	1
	None	8	10	8	8	18	16

^1^ The values in each cell may vary between 0 and 15 for individual years and between 0 and 30 for combined years; ^2^ BVD = Bovine viral diarrhoea; ^3^ IBR = Infectious bovine rhinotracheitis.

### 3.4. Calving Management

In HR herds (compared to LR herds) there was more likely to be a combination of calving units than a group calving unit (Table 2 and Table 6). In HR herds pregnant animals were more likely to be transferred to the calving unit within ≤12 h of calving than 12 to 24 h or more than 2 days before calving. In HR herds there was more likely to be 2 calving personnel. In addition, in HR herds calves were more likely to be resuscitated using a straw up the nostrils or water as a stimulant than no use of resuscitation. 

**Table 6:** Responses (No.) on calving management from farmers with a high rate (HR, n = 15) and low rate (LR, n = 15) of perinatal calf mortality.

Factor	Levels ^1^	HR 2010	LR 2010	HR 2012	LR 2012	HR & LR 2010	HR & LR 2012
Calving unit	Individual pen	8	7	7	7	15	14
	Group pen	2	7	2	7	9	9
	Outdoor pad	3	0	2	0	3	2
	Combination	2	1	4	1	3	5
Timing of transfer of animals to calving unit	≥2 days	2	7	2	7	9	9
	1 day	1	0	1	0	1	1
	12 h to <23.9 h	1	4	1	5	5	6
	0–11.9 h	10	4	10	3	14	13
	No transfer	1	0	1	0	1	1
Observation interval for calving signs	≥6 h	3	5	2	5	8	7
	4 h to 5.9 h	1	1	2	1	2	3
	2 h–3.9 h	9	9	9	9	18	18
	1 h–1.9 h	2	0	2	0	2	2
Time to assist heifers after legs appear	>2 h	2	4	2	4	6	6
	≤2 h	13	11	13	11	24	24
Time to assist cows after legs appear	>2 h	6	4	6	4	10	10
	≤2 h	9	11	9	11	20	20
Calving aid	Calf puller	15	15	15	15	30	30
	Other aid	1	3	1	3	4	4
Calving personnel (no.)	≥3	1	3	1	3	4	4
	2	6	2	6	2	8	8
	1	8	10	8	10	18	18
Calf revival	Water in ear	6	6	8	6	12	14
	Straw in nostrils	5	3	5	3	8	8
	Reviving products	4	0	4	0	4	4
	Other physical revival	6	8	8	8	14	16
	None	1	4	0	4	5	4
Milk fever control	Powdered minerals	8	6	8	5	14	13
	Therapy only	4	6	4	7	10	11
	Other control	3	3	3	3	6	6

^1^ The values in each cell may vary between 0 and 15 for individual years and between 0 and 30 for combined years.

Heifers and cows were more likely to be vaccinated against salmonellosis and IBR in 2012 than in 2010 (*P* < 0.05), irrespective of whether they were in HR or LR herds. No other management changes over time were evident from the questionnaire responses.

## 4. Discussion

The overall results are discussed first followed by the comparison between the case and control herds. As expected almost all herds had Holstein-Friesian heifers but a surprisingly high proportion of herds also had other dairy or crossbreed heifers, mainly Jerseys. This reflects a recent change within some herds in the Irish dairy industry (www.icbf.com) and internationally [21] towards crossbreeding. This is primarily in response to the recent decline in dairy herd fertility [22] as crossbred animals have superior reproductive performance. This change was also evident from the fact that in only 77% of the herds in 2012 were the heifers bred to Holstein-Friesian sires: the remainder were predominantly bred to either Aberdeen Angus (for calving ease) or Jersey sires (for calving ease, better milk solids and fertility). This trend was also apparent for the cows. It might be expected that with increased use of non-Holstein-Friesian genotypes bovine perinatal mortality may decline as dairy crossbred animals have been shown to have lower bovine perinatal mortality rates than Holstein-Friesians [23,24].

While heifers were fed grass silage pre-calving in all herds a relatively small proportion were offered supplementary feeds or straw. This may be because of the generally high BCS of the heifers pre-calving. Because of the association between trace element deficiencies, bovine perinatal mortality and calving problems, heifers were supplemented with trace elements (mainly by mineral powder) in the majority (90%) of herds. As with the heifers, all herds of cows were fed grass silage with only a relatively small proportion offered ration or straw. In all herds cows were supplemented with trace elements pre-calving, most commonly by mineral powder. Previous studies at this research centre have shown the benefits of this practice [25] and it has been recommended to Irish farmers. Thus the majority of farmers were implementing best practice.

The most commonly used vaccines (leptospirosis and salmonellosis: 86% of herds) in heifers reflect the perceived importance of these pathogens in causing abortions and weak calves in Irish dairy herds. However, while salmonella spp. are one of the most commonly diagnosed causes of abortion in Irish cattle herds [26], leptospira are detected much less frequently [27]. In recent years, with widespread publicity in the Irish farming media about BVD [28], the use of BVD vaccines has increased substantially from a low base [29]. Similar trends were found in cow vaccinations. Thus the majority of farmers were implementing best practice. The majority (70%) of farms had at least one farm dog and foxes were present on the majority (90%) of farms. Both of these species are intermediate hosts for *Neospora caninum*, a cause of perinatal calf mortality [30,31]. A small number of herds had a recent clinical history of neosporosis. However, the most common recent clinical problems relevant to bovine perinatal mortality in these herds were BVD (23% of herds) and salmonellosis (33%). 

Traditionally tie-up stalls in the 1960s and individual calving pens from the 1970s to the 1990s were the norm on Irish dairy farms, in the latter case due to their dual use as isolation pens for suspected cases of brucellosis. However, in this study only half of all herds had individual calving pens with a large proportion using group pens, outdoor pads or combinations of both. This apparent move away from individual pens may reflect the large herd sizes in this study (on average approximately 160 cows compared to the national average of 40 dairy cows/herd) [32], the eradication of brucellosis and attempts to save labour at a busy time of the year in these seasonally calving herds. In all study herds dedicated calving facilities were used. This contrasts with a recent Canadian study where such facilities were used in less than half of the herds surveyed [33].

A surprisingly small proportion of farmers (33%) moved their pregnant animals to the calving unit at least 24 h pre-calving. This may have been due to limited calving accommodation following gradual herd expansion over years. This early move allows animals to adapt to their new environment, social group and diet. On a large proportion (43%) of farms pregnant animals were moved to the calving unit between 6 h and 24 h pre-calving reflecting the detection of impending signs of calving such as relaxation of the sacrosciatic ligaments and/or colostrum dripping from the teats. A smaller proportion (20%) of farmers moved pregnant animals to the calving unit on the point of calving which may reflect limited observation of animals pre-calving or limited calving facilities. This ‘just in time’ calving movement protocol has become popular in North America and has recently been shown to have no detrimental effects on calving performance and is preferable to moving animals during stage one of calving [34].

The majority (73%) of farmers observed pregnant animals once they were in the calving unit for signs of impending calving every 4 h to 6 h. This is similar to the findings of a recent Canadian survey in dairy herds [33]. Given that stage one of calving may last this long this is adequate to detect the onset of this stage of calving but as stage two of calving may be on average, 45–90 minutes, this interval may miss this stage of calving completely. However, once the foetal hooves were observed the majority (63%) of farmers assisted heifers between 1 and 2 hours later. Similarly, cows were generally (73% of herds) assisted between 1 and 2 hours after the onset of stage two. This indicates that farmers were cognisant of avoiding prolonged calving. These findings are in agreement with an earlier Irish survey which found that the majority of farmers intervened within 2 hours of the onset of calving [35]. All farmers used a calving aid (‘calving jack’) and some used additional calving aids. Whilst such aids are common on Irish dairy farms [35], their use is regulated in some dairy industries, e.g., in Scandinavia and in the Netherlands.

Resuscitation techniques were used on the majority (86%) of farms, a figure comparable with that on US dairies (81%) [36]. The most common first-aid resuscitation techniques used (hypothermal stimulation, nasal stimulation and calf suspension) have been shown to induce a gasp reflex, improve blood acid-base balance and improve rectal temperature in newborn calves [37,38]. Control of milk fever was based on either providing mineral powder (composed of many macro and micro-nutrients including calcium, phosphorous and magnesium at concentrations designed to prevent hypocalcaemia) pre-calving or therapy of clinical cases reflecting the widespread use of mineral powder for prevention of other periparturient problems. 

Most of the management factors investigated here did not differ between case and control herds. This finding is congruent with the results of recent surveys on perinatal calf mortality in German [39] and in Icelandic herds [17]. Herdowners of high risk herds were more likely to use Je or JexHF sires on cows. This may be due to an attempt to reduce difficult calvings and bovine perinatal mortality in HF animals by crossbreeding with an easy calving breed such as the Jersey [40]. The lower BCS of heifers in the high risk herds may be a response to previous losses in heifers where over-conditioning was a contributory problem. Obesity in heifers is a risk factor for dystocia and calf loss [41,42]. However, the large ORs and wide CL95 for some of the BCS variables indicate that these outcomes could not be determined with confidence. 

Both heifers and cows in HR herds tended to be more likely to be vaccinated against leptospirosis, reflecting the importance attributed to this pathogen in causing abortions and bovine perinatal mortality [43] and probably also the results from laboratory confirmation of this pathogen causing abortions and perinatal mortality. 

Pregnant animals were moved to the calving accommodation earlier on LR compared to HR farms. This may facilitate adaption to the new environment, diet and social hierarchy, particularly in heifers, where such stresses can negatively impact calving performance [44]. The later transfer of pregnant animals to the calving unit in HR herds, during stage one of calving, is likely to lead to delay in the onset of stage two of calving, longer duration of stage two, more calving assistance, dystocia and stillbirths [34]. The greater use of a combination of different calving unit types in HR herds compared to LR herds may suggest attempts over time to provide better calving accommodation by adding to the existing maternity units with different designs. The greater use of group calving units in the LR herds may allow pregnant animals to express their natural pre-calving behaviours more easily as such units simulate the natural environment of the cow at pasture. Group calving accommodation was more common on LR farms which may reflect less stress of isolation in individual calving pens and more normal social interaction pre-calving [1]. There were no apparent differences between HR and LR farms in how calvings were supervised (observation intervals) or assisted (time to assist or use of calving aids). This is in contrast to an earlier study finding that the difference between a high and a low bovine perinatal mortality farm was the ability of the farm personnel to calve heifers successfully [19]. HR herds were more likely to have two people managing calvings but some LR herds had up to four people managing calvings. No information was gathered on the experience of the staff who managed calvings, a factor which may have a more important bearing on the quality of calving management than the number of personnel. Poor calving and calving pen management was identified in a survey of 28 Canadian dairy farms as a possible contributory factor in poor calf welfare [45].

Calves were more likely to be resuscitated using intranasal or hypothermal stimulation than no resuscitation in the HR herds. This may be due to ongoing calf losses in the HR herds. Both of the techniques listed, prodding a straw up the calf’s nostrils and pouring cold water in the ear or over the calf’s head, are commonly used revival practices on Irish farms [46] known to stimulate the gasp reflex and ultimately to improve gas exchange in the immediate postparturient period [37,38].

No apparent changes in the genotypes present on these farms over time were detectable which is not surprising given the short time frame of the study. No changes were apparent either in the feeding management of the heifers or cows pre-calving over this period. This may reflect the generally optimal feeding management adopted by these farmers and the previous changes they had made to prevent problems at calving. For both heifers and cows there was an increase in the number of herds vaccinated against salmonellosis and IBR over the three years. In the former case the incidence of clinical disease tends to have annual cycles [47] a peak of which may have occurred during these years which stimulated vaccination. In the case of IBR very few herds were vaccinated at the start of the study. However, in the intervening years greater publicity has been focused on this disease in the Irish farming media by both the pharmaceutical industry [48] and by Animal Health Ireland, a national body which provides advice on animal health management (http://www.animalhealthireland.ie/index.php). This probably influenced farmers to commence vaccination as no increase in clinical IBR was reported in these herds.

No changes in calving supervision, calving assistance, newborn calf care or milk fever control, were apparent over the three years. As these farmers appeared to generally implement best practice and these tended to be practices of habit and routine, changes were possibly unlikely to occur in this relatively short time frame.

## 5. Conclusions

This study demonstrates that some pre-calving and calving management factors differ between herds with high and low rates of bovine perinatal mortality. However, the majority of such factors were similar between these two herd categories. The findings here suggest that calving management factors are more important than pre-calving factors. For example, farmers who moved their cows to the calving area earlier pre-calving had lower rates of bovine perinatal mortality. The finding that calving management factors are of greater importance is a key conclusion and indicates where farmers and their veterinary practitioners need to focus both when investigating such problems and when attempting to reduce losses in herds with high rates of bovine perinatal mortality.
